# Binding Thermodynamics and Dissociation Kinetics Analysis Uncover the Key Structural Motifs of Phenoxyphenol Derivatives as the Direct InhA Inhibitors and the Hotspot Residues of InhA

**DOI:** 10.3390/ijms231710102

**Published:** 2022-09-03

**Authors:** Qianqian Zhang, Jianting Han, Yongchang Zhu, Shuoyan Tan, Huanxiang Liu

**Affiliations:** 1 Faculty of Applied Science, Macao Polytechnic University, Macao, China; 2 School of Pharmacy, Lanzhou University, Lanzhou 730000, China; 3 College of Chemistry and Chemical Engineering, Lanzhou University, Lanzhou 730000, China

**Keywords:** InhA direct inhibitors, phenoxyphenol derivatives, hotspot residues, pharmacophore model, molecular dynamics simulation, dissociation pathway, residence time

## Abstract

Given the current epidemic of multidrug-resistant tuberculosis, there is an urgent need to develop new drugs to combat drug-resistant tuberculosis. Direct inhibitors of the InhA target do not require activation and thus can overcome drug resistance caused by mutations in drug-activating enzymes. In this work, the binding thermodynamic and kinetic information of InhA to its direct inhibitors, phenoxyphenol derivatives, were explored through multiple computer-aided drug design (CADD) strategies. The results show that the van der Waals interactions were the main driving force for protein–ligand binding, among which hydrophobic residues such as Tyr158, Phe149, Met199 and Ile202 have high energy contribution. The AHRR pharmacophore model generated by multiple ligands demonstrated that phenoxyphenol derivatives inhibitors can form pi–pi stacking and hydrophobic interactions with InhA target. In addition, the order of residence time predicted by random acceleration molecular dynamics was consistent with the experimental values. The intermediate states of these inhibitors could form hydrogen bonds and van der Waals interactions with surrounding residues during dissociation. Overall, the binding and dissociation mechanisms at the atomic level obtained in this work can provide important theoretical guidance for the development of InhA direct inhibitors with higher activity and proper residence time.

## 1. Introduction

The enoyl-acyl carrier protein reductase (InhA) is a key enzyme in catalyzing fatty acid synthesis, which is essential for the survival of mycobacteria. Studies have shown that inhibiting the function of InhA protein will block the synthesis of mycolic acid, thereby destroying the formation of bacterial cell walls and further leading to morphological changes and cell lysis [1]. As prodrugs, isoniazid and ethionamide are activated by KatG/EthA to form adducts with NADH, and then the adduct acts on InhA to inhibit its protein function [2,3]. However, mutations in the KatG/EthA genes confer severe resistance to isoniazid and ethionamide [4]. In contrast, the direct inhibitors of InhA can skip the drug activation step and avoid the drug resistance problems. Therefore, the study of direct inhibitors against InhA target has attracted widespread interest.

In recent years, a series of phenoxyphenol derivatives designed based on the antibacterial drug triclosan (TCL) showed high binding affinity to InhA [5,6,7]. In particular, the derivatives have longer residence time than TCL and can act on the InhA target for a long time [6,8]. Additionally, some direct inhibitors, including thiazoles, pyridines and carboxamides [9,10,11], etc., have also been discovered through high-throughput screening and fragment-based drug design. Unfortunately, none of the inhibitors have entered clinical studies. So, it is very meaningful to understand the interaction essence between InhA and its direct inhibitors to provide the valuable information of future design and modification of InhA direct inhibitors.

In modern drug discovery, accurate evaluation of thermodynamic and kinetic information of drug–target interactions can provide a useful theoretical basis for understanding how drugs and their targets interact and how to improve drugs’ efficacy [12,13]. With the development of computer-aided drug design (CADD), the binding free energy [14,15,16] and residence time [17,18] of protein–ligands can be accurately predicted by computational methods. Compared to traditional experiments, CADD strategy is more time-efficient, labor-saving and cost-effective, allowing the prediction of thermodynamic and kinetic information for many molecules in a short period of time.

In this paper, the binding thermodynamics of TCL and its five derivatives (PT70, PT91, PT119, PT501 and PT506) with InhA was first explored by classic molecular dynamics (MD) simulations. The binding free energy calculated by the molecular mechanics–generalized Born surface area (MM–GBSA) [19] showed that these inhibitors bound to InhA mainly through the van der Waals interaction. Residue energy decomposition revealed that residues such as Phe149, Tyr158, Met199 and Ile202 had higher energy contributions to the binding of inhibitors. In addition, the conformation of the substrate binding loop (H6) and H7 directly affected the binding affinity of the inhibitors. Secondly, pharmacophore model analysis further showed that the common structural features of this class of inhibitors include a hydrogen bond acceptor group, a hydrophobic group and two aromatic centers. The results of tau random accelerated MD (τRAMD) [18] simulations indicated that the six inhibitors mainly have two different dissociation pathways, and the predicted order of residence time was consistent with the experimental order. Meanwhile, steered MD [20] simulations further characterize the intermediate states of each inhibitor during the dissociation process.

## 2. Results and Discussion

### 2.1. Van der Waals Interactions Are the Main Driving Force for the Binding of InhA Inhibitors

**Figure 1:**
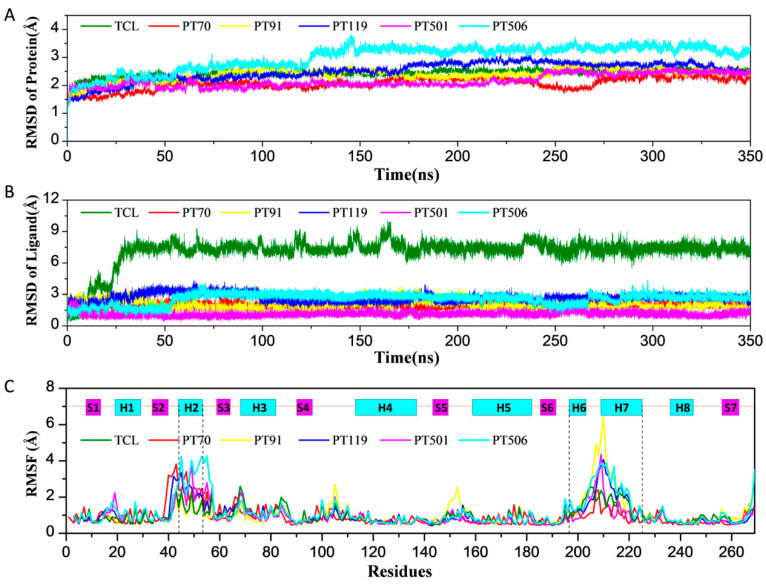
(**A**) Time evolution of the RMSD values of the protein. (**B**) Time evolution of the RMSD values of the heavy atoms of ligands. (**C**) The RMSF values of the protein.

The root-mean-square deviations (RMSDs) value can be used to examine the deviation between the target coordinate set and the reference coordinate set during the simulation process, which reflects the positional change of the molecular structure over time. The fluctuations of RMSD can also characterize the stability and convergence of the system. Here, the RMSD values of protein and ligand over time were monitored during the simulation. It can be seen from Figure 1A that after 150 ns, the protein RMSD fluctuations in each system were all within 1.0 Å, indicating that the system tended to be stable. As shown in Figure 1B, all ligands were also relatively stable after 50 ns (fluctuated within 1.0 Å). The RMSD of TCL changed greatly at about 30 ns (from 1.0 Å to 8.0 Å), which indicated that the binding position of TCL may have changed. In addition, the root-mean-square fluctuations (RMSFs) of proteins were also monitored. From Figure 1C, we can see that among the six systems, the H6 and H7 were more flexible than the other domains. The α-helix of H6 and H7 are located around the binding pocket, which directly affects the stability of inhibitor binding. The flexibility of H6 and H7 was the smallest in PT70 system, which may be the reason for the best binding affinity of PT70. 

To predict the binding affinity between these inhibitors and InhA, the equilibrated trajectories were used to calculate the binding free energies by the MM–GBSA method. As shown in Table 1, the binding free energies of PT70, PT91, PT119, PT501, TCL and PT506 to InhA were −32.84 kcal/mol, −32.23 kcal/mol, −32.27 kcal/mol, −32.09 kcal/mol, −28.34 kcal/mol and −28.11 kcal/mol, respectively. By comparing the contributions of various energy terms, it can be seen that the van der Waals interaction energies were much higher than the electrostatic interaction energies, indicating that the van der Waals interaction was the main driving force for the binding of inhibitors. In addition, the order of the enthalpy changes was basically consistent with the order of the Ki values measured experimentally. 

### 2.2. Critical Roles of Hotspot Residues and the Conformation of H6/H7 on the Binding of InhA Inhibitors 

**Figure 2:**
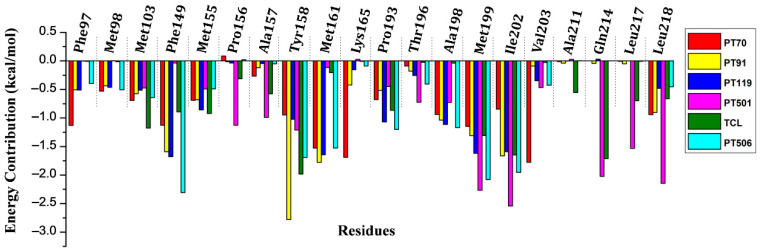
The energy contribution spectrum of key residues to the binding of different InhA direct inhibitors.

**Figure 3:**
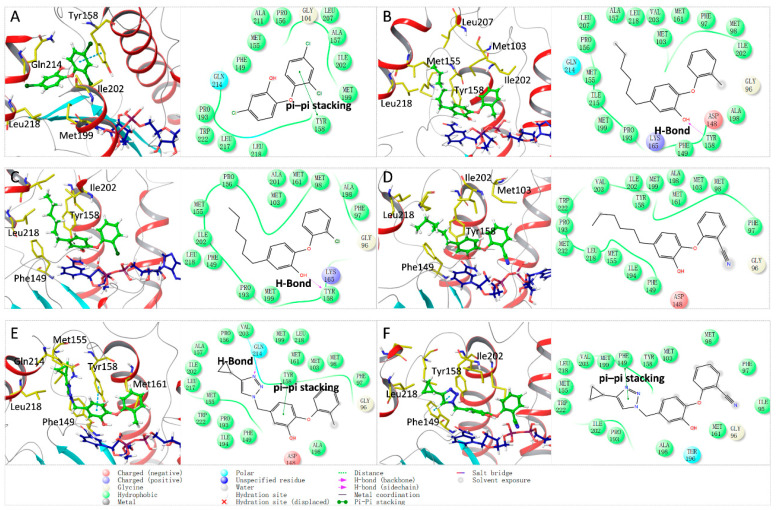
The detailed 3D and 2D binding mode for the representative conformation (**A**) TCL, (**B**) PT70, (**C**) PT91, (**D**) PT119, (**E**) PT501 and (**F**) PT506.

To further identify hotspot residues for the binding of these inhibitors to InhA, residue energy decomposition was carried out. As shown in Figure 2, the residues with large energy contributions mainly included Phe149, Tyr158, Met161, Met199, Ile202 and Leu218. To analyze the relationship between the residue energy contribution and the binding mode, principal component analysis was performed, and the free energy landscapes were plotted based on the top two principal components (Figure S1). It can be seen that the conformational distribution of each system was relatively concentrated, tending to form a single conformational state. The structures located at the lowest energy well were selected as the representative conformations of each system and the detailed binding modes between them and InhA were shown in Figure 3. It can be seen that the residue Tyr158 can not only form pi–pi stacking with the benzene rings on TCL and PT501 molecules, but also form hydrogen bonds with the hydroxyl groups on PT70 and PT91 molecules. Therefore, the interaction between the residue Tyr158 and the inhibitor is very important for the binding of inhibitors. Moreover, the benzene rings and hydrophobic tails of TCL, PT70, PT91 and PT119 can also form van der Waals interactions with residues Phe149, Phe97, Met103, Met155, Tyr158, Met161, Met199 and Ile202. In the PT501 system, the N atom of the triazole ring can act as a hydrogen bond acceptor to form a hydrogen bond with Gln214. The triazole and cyclopropane substituent of PT501 protruded into the hydrophobic pocket formed by Met155, Leu217, Ile202, Ala157 and Pro156 (Figure 3E). The above residues were also critical to the binding of PT501 and InhA (Figure 2). The triazole ring of PT506 can form pi–pi stacking interaction with Phe149. In addition, the triazole ring and cyclopropane substituents also extended into the hydrophobic pocket formed by Met199, Val203, Leu218, Met155, Trp222, Ile202 and Pro193. 

Previous studies have shown that H6 and H7 were critical for inhibitor binding and the above RMSF analysis also indicated that the flexibility of H6 and H7 was quite different among different inhibitors. Therefore, to further explore the conformational differences of H6 and H7, the H6 and H7 structure of PT70 were superimposed with the other systems, respectively (Figure S2). Compared to PT70 system, the α-helix structure of H6 was converted into a loop structure in the TCL system, and the helix structure of H7 also underwent a conformational change. The binding conformation of PT91 was similar to that of PT70, with both their hydrophobic tails extending to the hydrophobic sub-binding pocket above. However, the α-helix of H6 in the PT91 system was also converted to a loop structure, which increased the flexibility of the binding pocket and reduced the binding affinity of PT91 to InhA. In the PT501 system, the conversion of the helix structure of H6 and H7 to loop was more obvious. Additionally, we found the binding conformations of cyano substituents of PT119 and PT506 were similar. The conformations of H6 and H7 in both PT119 and PT506 systems underwent large shifts, which ultimately exposed their binding pockets to solvent, thereby reducing their binding stability to InhA.

### 2.3. Pharmacophore Model Analysis Reveals the Structural Motifs of Phenoxyphenol Derivatives as InhA Direct Inhibitors

**Figure 4:**
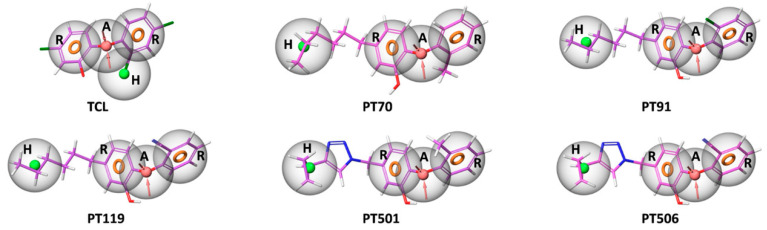
Alignment between the multiple ligand-based AHRR pharmacophore model and each inhibitor. The ligands are shown as purple sticks. Orange ring: aromatic ring (R), pink ball: hydrogen bond acceptor (A), green ball: hydrophobic group (H).

The structural features of phenoxyphenol derivative inhibitors were further explored by the pharmacophore model. As shown in Table 2, there are three pharmacophore models based on multiple-ligand generation: AHRR, HHRR and AHHRR. Among them, the AHRR model has the highest AUC value (0.89), which contained a hydrogen bond acceptor (A), a hydrophobic group (H) and two aromatic rings (R). Furthermore, all six inhibitors fit the AHRR pharmacophore profile. The alignment of the AHRR pharmacophore model with each inhibitor is shown in Figure 4. Therefore, the AHRR pharmacophore model can best display the common structural features of phenoxyphenol derivative inhibitors. 

From the pharmacophore model, it can be seen that the aromatic ring and hydrophobic center of these inhibitors play key roles in protein–ligand binding, further supporting that van der Waals interactions are the main driving force for the ligand binding. The benzene rings and triazole ring on the inhibitors can generate an aromatic center, which can further form pi–pi stacking interaction with Tyr158 or Phe149 residues. At the same time, the hydrophobic tails of the inhibitors can generate a hydrophobic center, which penetrated deep into the hydrophobic pocket of the receptor. On the other hand, the pharmacophore model also shows that there are fewer polar centers (such as hydrogen bond acceptors and hydrogen bond donors) in the inhibitors, so the polar interactions between the ligands and the acceptors are relatively weak. Therefore, the introduction of polar groups into the inhibitor can be considered in the future molecular design, which can increase the solubility of the molecule on the one hand and can also enhance the polar interaction between the molecule and the target. At the same time, virtual screening based on the pharmacophore model can also further search for analogs with more possible polar groups.

### 2.4. The Order of Residence Time Predicted by τRAMD Is Consistent with the Experiment

**Figure 5:**
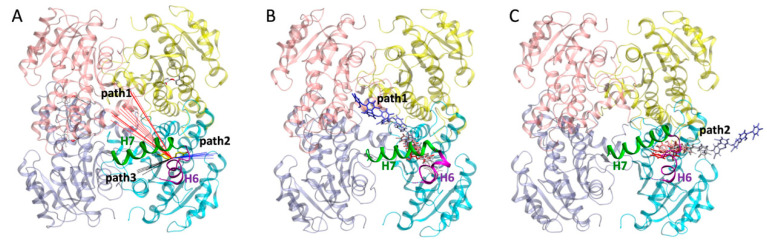
(**A**) Schematic diagram of three possible dissociation pathways (path1 to path3). (**B**) Schematic diagram of the dissociation path of TCL along path1. (**C**) Schematic diagram of the dissociation path of TCL along path2.

To explore the dissociation kinetics of InhA inhibitors, each inhibitor was dissociated using the tau random acceleration molecular dynamics simulation (τRAMD) method. For the six inhibitors studied in this work, we found a total of three possible dissociation pathways (as shown in Figure 5A). Among them, in the path1 channel, the inhibitors dissociated along the H7 side, and in the path2 channel, they dissociated along the H6 side, while in the path3 channel, the inhibitors dissociated from the gap formed by H6 and H7. Furthermore, the dissociation orientations of the 120 trajectories for each inhibitor were classified and counted (as shown in Table S1). The results showed that the two most dominant dissociation pathways adopted by the six inhibitors were path1 and path2, while path3 was rarely present. It can be seen from Table S1 that the number of dissociated trajectories of TCL according to path1, path2 and path3 were 50, 69 and 1, respectively. It can be speculated that TCL is relatively small and can dissociate from both path1 and path2 channels (Figure 5B,C). For the PT70, PT91 and PT501, their main dissociation pathways were mainly according to the path1 channel, while PT119 and PT506 were mainly along the path2 channel (Table S1 and Figure S3). 

Furthermore, we averaged the dissociation times of 120 simulated trajectories to predict the residence times for each inhibitor (as shown in Table 3). The residence times of TCL, PT91, PT70, PT119, PT501 and PT506 predicted by τRAMD were 33.9 ps, 91.4 ps, 177.4 ps, 386.5 ps, 518.9 ps and 2529 ps, respectively. Compared to the experimental value, the predicted residence times were much shorter (only at the ps level) due to the applied external force in τRAMD. However, the order of the predicted residence times of the six molecules was consistent with the order that was measured experimentally. In addition, τRAMD also predicted the residence time of TCL that cannot be experimentally monitored due to its too-fast dissociation. From the predicted residence time, we found that inhibitors containing cyano substitutions have longer residence times, such as PT119 and PT506. Through the analysis of the binding mode, we found that the N atom of the cyano group of PT119 and PT506 can form hydrogen bonds with the oxygen atoms of the pyrophosphate region of NADH (as shown in Figure S4), which was not shown in PT70, PT91 and PT501 systems. Therefore, PT119 and PT506 required longer time to overcome the hydrogen bonds between them and NADH during the dissociation process. Therefore, the cyano group substitution may prolong the residence time of the molecule, which is information that can guide the design of the inhibitor of InhA with long residence time.

### 2.5. Steered MD Identifies the Intermediate States during the Dissociation Process

**Figure 6:**
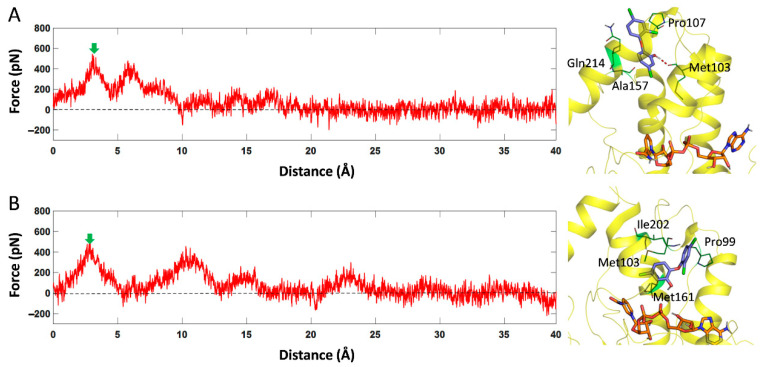
The force profile of TCL over the reaction coordinate along path1 channel (**A**) and path2 channel (**B**). The structures at the maximum force (green arrow) were extracted and displayed on the right side of the picture.

To further explore the key residues and interactions between these inhibitors and InhA during the dissociation process, steered MD simulations were performed. As shown in Figure 6, the position of the maximum force of TCL in the two dissociation pathways was about 3 Å. The results show that during the dissociation of TCL along path1, the hydroxyl group on its benzene ring can form a hydrogen bond with the backbone oxygen atom of Met103. In addition, hydrophobic residues such as Gln214, Pro107 and Ala157 at the dissociation exit can also form van der Waals interactions with the benzene ring, thereby hindering the dissociation of TCL here. However, when TCL dissociated along path2, the benzene ring of TCL just fell into the hydrophobic pocket formed by Ile202 on H6 and residues, such as Met103, Met161 and Pro99, at the exit.

**Figure 7:**
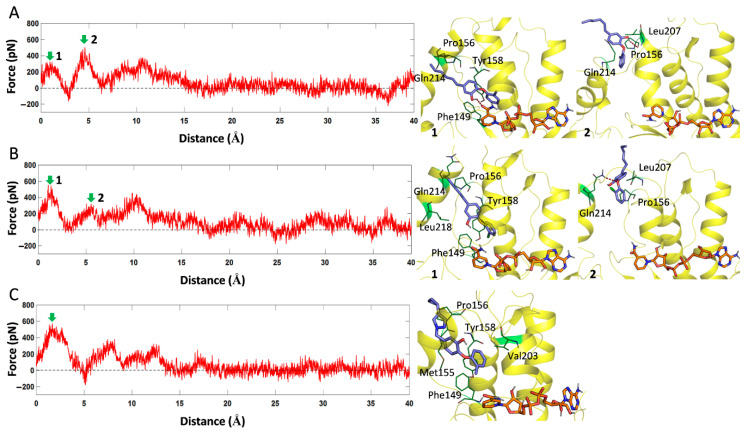
The force profiles of PT70 (**A**), PT91 (**B**) and PT501 (**C**) over the reaction coordinate along path1 channel. The structures at the maximum force (green arrow) were extracted and displayed on the right side of the picture. The number “1” corresponds to the first intermediate state in the dissociation process, and the number “2” corresponds to the second intermediate state.

For PT70 and PT91, they have two different intermediate states during dissociation along the path1 channel (as shown in Figure 7). For the PT70 system, the first intermediate state indicated that the hydroxyl group of PT70 can form a hydrogen bond with the oxygen atom of NADH. In the second intermediate state, the hydrophobic tail of PT70 had dissociated out of the pocket, and the hydrogen bond was formed between the hydroxyl group and the oxygen of Pro156. Meanwhile, PT70 also formed van der Waals interactions with surrounding residues such as Tyr158, Phe149, Pro156, Leu207 and Gln214 during dissociation process (Figure 7A). For PT91, its first intermediate state was mainly hindered by the van der Waals interaction of some hydrophobic residues including Tyr158, Phe149, Pro156, Leu218 and Gln214. In the second intermediate state, the hydrophobic tail of PT91 also had dissociated like PT70, while its ether oxygen atom can form a hydrogen bond with the Gln214. As for PT501, it was also hindered by hydrophobic residues such as Tyr158, Phe149, Pro156, Met155 and Val203 during dissociation along the path1 channel. However, since the volume of the triazole ring and cyclopropane on the PT501 molecule is larger than that of the hydrophobic tail of PT70 and PT91, it experienced greater resistance during the dissociation process. Therefore, the dissociation time of PT501 was also longer. For PT119 and PT506, their main dissociation pathways were along the path2 channel. 

**Figure 8:**
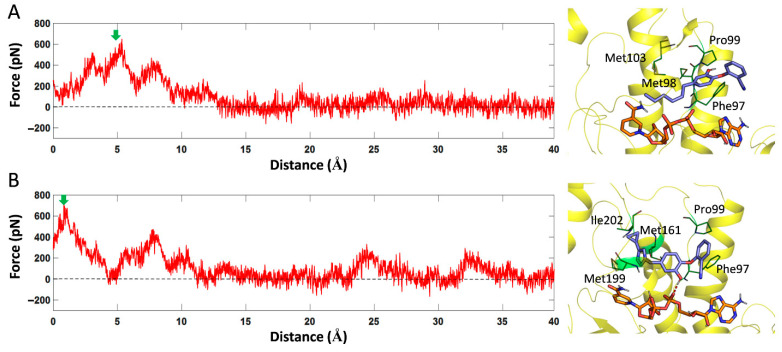
The force profiles of PT119 (**A**) and PT506 (**B**) over the reaction coordinate along path2 channel. The structures at the maximum force (green arrow) were extracted and displayed on the right side of the picture.

As shown in Figure 8A, PT119 can form van der Waals interactions with residues such as Phe97, Met98, Pro99 and Met103 during dissociation. Although the dissociation direction of PT506 molecule was consistent with that of PT119, the structure of PT506 was larger than that of PT119, therefore it can form more interaction with surrounding residues, such as with Ile202, Met199, Met161, Pro99 and Phe97 (Figure 8B). In addition, the hydroxyl group of PT506 can also form hydrogen bonds with the oxygen atom of NADH. Therefore, the dissociation time of PT506 was much longer than that of the PT119 molecule.

## 3. Materials and Methods 

### 3.1. System Preparation 

**Figure 9:**
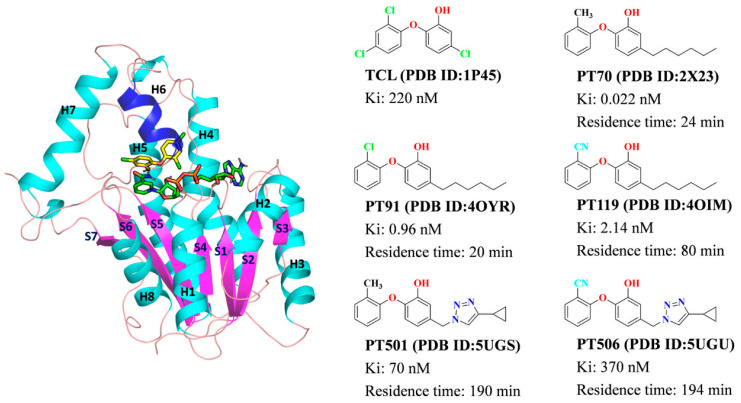
The binding mode of InhA with TCL (**left**); the chemical structures of the studied six inhibitors as well as their crystal structures with InhA: TCL (1P45 [29]), PT70 (2X23 [30]), PT91 (4OYR [6]), PT119 (4OIM [5]), PT501 and PT506 (5UGS, 5YGU [31]) (**right**). The β-sheet (S1–S7) was colored with magenta cartoon, the α-helix (H1–H8) was colored with cyan cartoon and the loop region was colored with pink.

The complex of InhA with each inhibitor was extracted from the PDB database (https://www.rcsb.org/) (accessed on 2 September 2021) (as shown in Figure 9). First, the Protein Preparation Wizard module of Schrödinger 2021 [21] was used to complete the missing side chains of protein and assign the protonation states of histidine at pH 7.0 automatically. Then, Gaussian 16 [22] software was used to calculate the RESP charges of the inhibitors at the Hartree–Fock 6–31G* level [23]. After that, RESP charge fitting was performed using the antechamber program in Amber 20 software (Version 2020, Case, D. A. et al., University of California, San Francisco, CA, USA) [24]. The parmchk module further generated the parameters for inhibitors. The tleap module was employed to generate topology and coordinate files for each complex. In addition, the GAFF [25] and ff19SB [26] force fields were used to describe inhibitors and proteins, respectively. Subsequently, each system was placed in a TIP3P water box [27]. The complex was set to be 10 Å away from the box edge, and periodic boundary conditions were employed to avoid boundary effects. Finally, the counter ions (Na+) were added to each system to neutralize the entire system [28].

### 3.2. Classic Molecular Dynamics Simulations

The PMEMD module [32] of Amber 20 was used to minimize the energy of each system to eliminate atomic collisions. With the complex restrained, each system was heated from 0 K to 310 K in three steps under the NVT ensemble [33]. Subsequently, equilibrium simulation was performed for each system. Finally, 350 ns production simulations were performed using the GPU version of Amber 20 [24] under the NPT ensemble [34]. During the simulations, the particle mesh Ewald (PME) algorithm [35] was used to treat long-range electrostatic interactions, and the SHAKE algorithm [36] was used to limit the vibrations of all covalent bonds involving hydrogen atoms. A structure was saved every 5 ps. Each system contains approximately 110,000 atoms.

### 3.3. Pharmacophore Modeling

A pharmacophore is a collection of chemical features and spatial properties necessary for ligand recognition by biological macromolecules. In this work, the pharmacophore models based on multiple ligand (6 inhibitors studied in this paper) were constructed by Schrödinger 2021 [21]. In addition, the discriminative ability of the pharmacophore model was also evaluated. The active set contained 13 active phenoxyphenol derivatives listed in literature [37] (not the same as the studied 6 inhibitors). The decoy set (including 2300 compounds) was downloaded from the DUD–E (Directory of Useful Decoys) [38]. Next, parameters such as phase hypo score, ROC (receiver operating characteristic), EF1% (enrichment factors) and AUC (area under the curve) were used to evaluate the pros and cons of the model. A pharmacophore model with a higher AUC value can more accurately find active inhibitors from the screened compounds.

### 3.4. Tau Random Acceleration Molecular Dynamics Simulation

The random acceleration molecular dynamics (RAMD) [39] simulation is a method developed by Lüdemann et al. to explore possible dissociation pathways for ligands. Based on this method, Kokh et al. developed τRAMD [18] to predict the residence time for a range of HSP90 inhibitors. τRAMD simulations require neither prior knowledge about protein–ligand binding nor extensive parameter fitting. The only parameter is the magnitude of the random force that affects the simulation time. In this paper, the τRAMD simulations were performed in NAMD program [40]. Firstly, 10 ns conventional MD simulations were performed to generate different initial structure and velocity for τRAMD simulation. Six structures were extracted from the trajectory (one frame every 2 ns) for τRAMD simulations. In addition, the magnitude of the random force was set to be 16 kcal/mol. To increase the repeatability of the simulations, 20 parallel dissociation simulations (with random force direction changes) were performed for each structure. Finally, a total of 120 dissociation trajectories were obtained for each system.

### 3.5. Steered Molecular Dynamics Simulation

The steered molecular dynamics (SMD) simulation [20] was used to explore the intermediate states during the dissociation process. In SMD, the reaction coordinates were set according to the dissociation pathway obtained in τRAMD simulation. In order to ensure that the springs in the SMD simulation were hard springs, we performed parameter corrections. The stretching speed was set to 0.0008 Å/ps, and the elastic coefficient was set to 10, 20, 30 and 40 kcal/mol·A^−2^, respectively. The results showed that when the elastic coefficient was set to 20 kcal/mol·A^−2^ (taking the PT70 system as an example), the requirement of a hard spring can be achieved (Figure S5). In addition, other systems also meet the hard spring requirement at a stretching speed of 0.0008 Å/ps and an elastic coefficient of 20 kcal/mol·A^−2^ (Figure S6). To avoid translation and rotation of the protein during the simulation, some residues away from the binding pocket were restrained. The simulations were performed in NAMD program.

### 3.6. MM–GBSA Calculation

The binding affinity of proteins–ligands has always been a research hotspot in the process of drug design. MM–GBSA is one of the important methods to calculate the binding free energy between proteins–ligands [41]. The basic principle of MM–GBSA is as follows:

(1) $$∆G_{\mathrm{bind}}=G_{\mathrm{complex}\;}-G_{\mathrm{receptor}}-G_{\mathrm{ligand}}$$

where $∆G_{\mathrm{bind}}$ is the binding free energy. $G_{\mathrm{complex}\;,}G_{\mathrm{receptor}}$ and $G_{\mathrm{ligand}}$ represent the free energies of complex, protein and ligand, respectively, which can be estimated by the following equations:

(2) $$G=E_{\mathrm{gas}}+{\;G}_{\mathrm{sol}}-TS$$

(3) $$G_{\mathrm{sol}}=G_{\mathrm{GB}}+G_{\mathrm{SA}}$$

(4) $$G_{\mathrm{SA}}=\gamma \times \mathrm{SASA}+\beta$$

where *E*_gas_ is the gas–phase energy. $G_{\mathrm{sol}}$ is solvation free energy, which can be calculated by solving the GB equation [42]. *G*_SA_ was estimated by the solvent-accessible surface area [43] determined using a water probe radius of 1.4 Å. The surface tension constant *γ* was set to 0.0072 kcal/mol·Å^2^ and β was set to 0 kcal/mol. *T* and *S* are the temperature and entropy. 

## 4. Conclusions

In this work, the multiple strategies (including classic MD simulation, MM–GBSA, pharmacophore model, τRAMD and steered MD simulation) were employed to investigate the binding thermodynamic and dissociation kinetics information of TCL and its five derivatives (PT70, PT91, PT119, PT501 and PT506) to InhA. The obtained results indicated that the residues such as Phe149, Tyr158, Met161, Met199 and Ile202 had high energy contributions to the binding of inhibitors. Compared with the PT70 system, the larger conformational changes in the α-helical structures of H6 and H7 in other systems increased the flexibility of the pocket and exposed the binding pocket to solvent, thereby reducing the binding affinity of the inhibitors. Furthermore, the pharmacophore model revealed that the active phenoxyphenol derivatives generally contain aromatic centers, hydrophobic centers and a hydrogen bond acceptor. The result also indicated that the aromatic centers and hydrophobic centers of these inhibitors can form pi–pi stacking interaction with Tyr158, Phe149 and van der Waals interactions with other hydrophobic residues of InhA. Although the oxygen atoms and hydroxyl substituents on the inhibitors could form hydrogen bonds with InhA, the hydrogen bond strength was relatively weak. Therefore, it can be considered to extend the substituent side chain and add more polar groups to establish stronger hydrogen bond interactions.

In addition, the dissociation pathways and residence times of these inhibitors were also predicted by τRAMD simulations. The results of τRAMD simulations showed that the two main dissociation channels were path1 (along the H7 direction) and path2 (along the H6 direction). Furthermore, the order of the predicted residence times of the inhibitors by τRAMD simulation was consistent with the experimental order. The steered MD simulations also identified that hydrophobic interactions (such as with Phe149, Met155, Tyr158, Ile202, Val203 and Leu207, etc.) and some hydrogen bond interactions (such as TCL and Met103, PT70 and Pro156, as well as PT91 and Gln214 residues) played important roles in the dissociation process. In conclusion, this work can deepen our understanding for the binding mode and dissociation process of InhA with phenoxyphenol derivatives. At the same time, the identified hotspot residues of InhA and the structural motif of phenoxyphenol derivatives inhibitors can also provide important guidance for the development of inhibitors in the future. 

## Figures and Tables

**Table 1:** The binding free energy and the contribution of different energy terms (kcal/mol).

Energy	PT70	PT91	PT119	PT501	TCL	PT506
Δ*E*_ele_	−9.00 ± 0.09	−17.01 ± 0.10	−6.81 ± 0.11	−11.72 ± 0.19	−4.71 ± 0.08	−7.56 ± 0.12
Δ*E*_vdw_	−39.18 ± 0.08	−39.63 ± 0.07	−40.74 ± 0.07	−42.63 ± 0.08	−34.01 ± 0.06	−38.94 ± 0.07
Δ*E*_MM_	−48.18 ± 0.11	−56.64 ± 0.11	−47.55 ± 0.12	−54.35 ± 0.19	−38.72 ± 0.10	−46.49 ± 0.16
Δ*G*_SA_	−5.58 ± 0.006	−5.70 ± 0.006	−5.82 ± 0.005	−5.67 ± 0.007	−4.38 ± 0.004	−5.12 ± 0.007
Δ*G*_GB_	20.91 ± 0.06	30.11 ± 0.09	21.09 ± 0.09	27.93 ± 0.15	14.77 ± 0.07	23.51 ± 0.10
Δ*G*_sol_	15.34 ± 0.06	24.42 ± 0.08	15.27 ± 0.09	22.26 ± 0.15	10.38 ± 0.07	18.38 ± 0.10
Δ*H*_bind_	−32.84 ± 0.08	−32.23 ± 0.07	−32.27 ± 0.07	−32.09 ± 0.08	−28.34 ± 0.07	−28.11 ± 0.09
Ki (nM)	0.022	0.96	2.14	70	220	370

**Table 2:** Validation of the multiple ligand-based pharmacophore models.

Pharmacophore	Phase Hypo Score	ROC	EF1%	AUC
AHRR_1	1.01	0.83	60.43	0.89
AHRR_3	0.93	0.67	60.43	0.77
HHRR_2	0.71	0.53	30.21	0.74
AHHRR_2	0.71	0.38	30.21	0.68

(A) hydrogen bond acceptor; (H) hydrophobic group; (R) aromatic ring. Phase Hypo score, ROC (Receiver Operating Characteristic), EF1% (Enrichment Factors) and AUC (Area Under the Curve) are the evaluation parameters for the quality of the models.

**Table 3:** Residence time ranking of InhA inhibitors predicted by τRAMD.

Ligand	Experiment (min)	τRAMD (ps)	Path
TCL	-	33.9	path1 or path2
PT91	20	91.4	path1
PT70	24	177.4	path1
PT119	80	386.5	path2
PT501	190	518.9	path1
PT506	194	2529.0	path2

## Data Availability

Not applicable.

## Supplementary Materials

The following supporting information can be downloaded at: https://www.mdpi.com/article/10.3390/ijms231710102/s1.
