# Supplementary material for: Binding Thermodynamics and Dissociation Kinetics Analysis Uncover the Key Structural Motifs of Phenoxyphenol Derivatives as the Direct InhA Inhibitors and the Hotspot Residues of InhA
Source: Int J Mol Sci. 2022 Sep 3;23(17):10102. doi: 10.3390/ijms231710102 (PMC9456180; doi:10.3390/ijms231710102)
Supplement: Supplementary file 1 [file ijms-23-10102-s001.zip › ijms-1863046-supplementary.pdf]

Supplementary Material

Binding Thermodynamics and Dissociation Kinetics Analysis Uncover the Key Structural Motifs of Phenoxyphenol Derivatives as the Direct InhA Inhibitors and the Hotspot Residues of InhA

Qianqian Zhang[1,2], Jianting Han[3], Yongchang Zhu[3], Shuoyan Tan[3], Huanxiang Liu[1]*

[1]Faculty of Applied Science, Macao Polytechnic University, Macao, SAR, China

[2]School of Pharmacy, Lanzhou University, Lanzhou 730000, China

[3]College of Chemistry and Chemical Engineering, Lanzhou University, Lanzhou, China

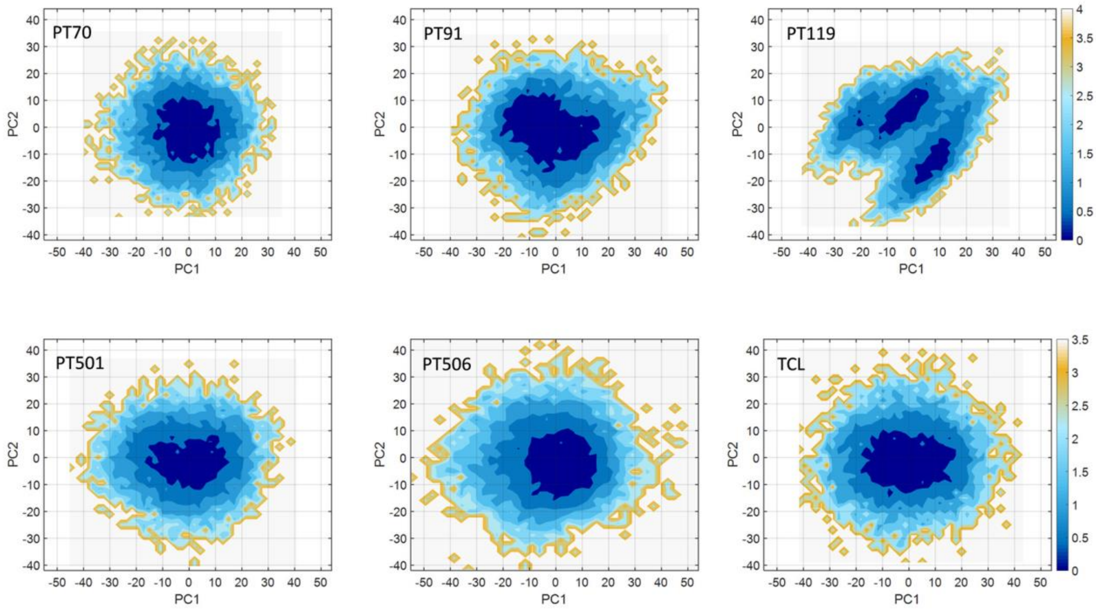

Figure S1. The free energy landscapes plotted based on the top two principal components for each system.

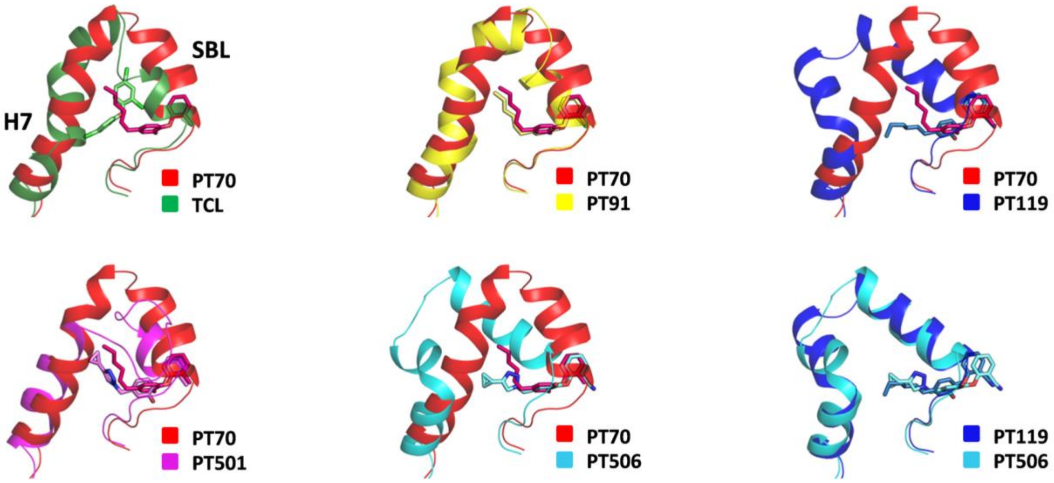

Figure S2. Superposition of H6 and H7 in the PT70 system with the other five systems, respectively.

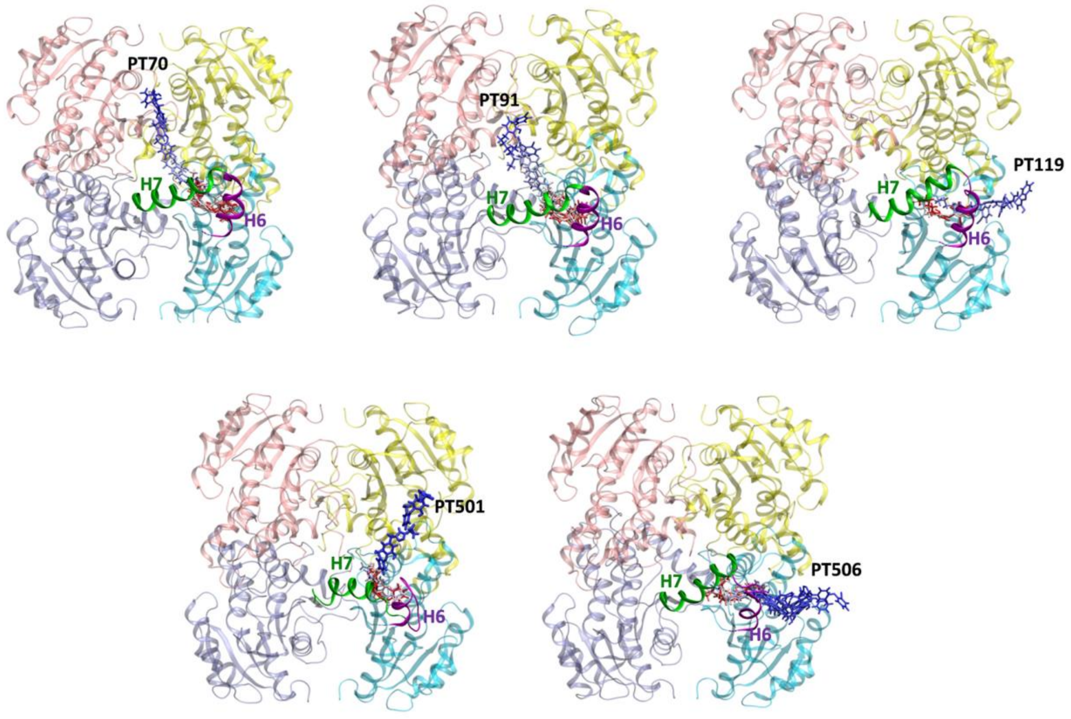

Figure S3. Schematic diagram of the dissociation pathways of PT70, PT91, PT119, PT501 and PT506 along their major dissociation channel.

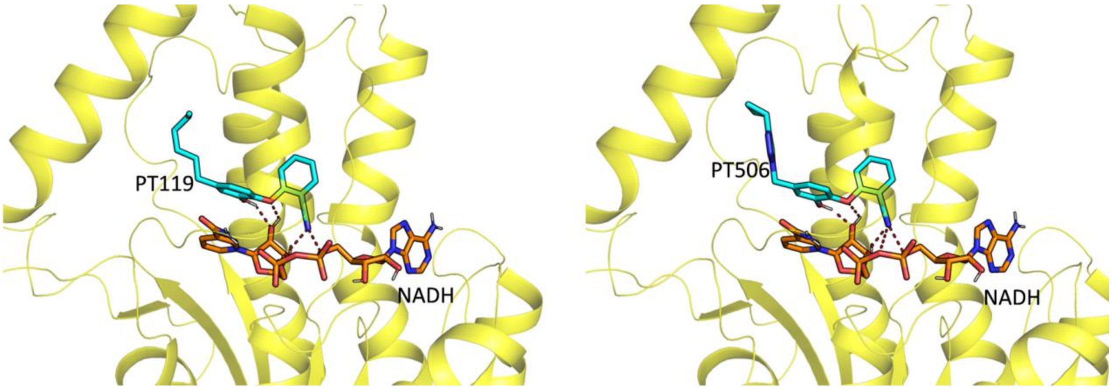

Figure S4. The cartoon diagram of the hydrogen bond formed between the cyano groups of PT119, PT506 and NADH.

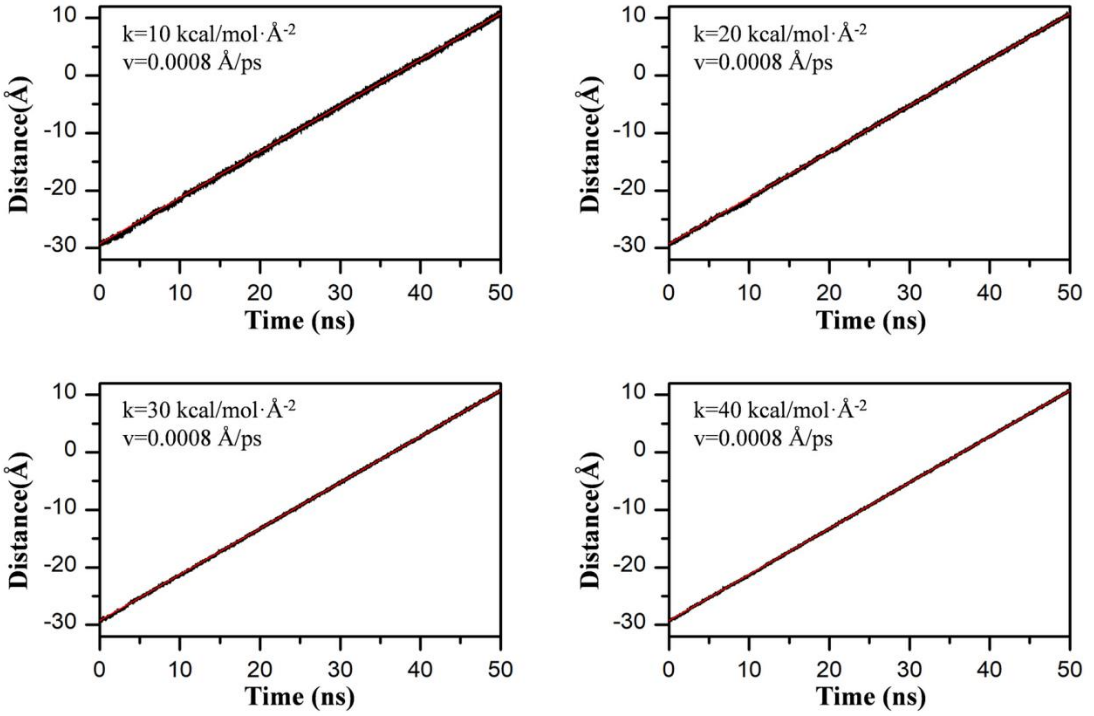

Figure S5. Variation of the distance between the ligand and the center of mass of the binding pocket versus simulation time for PT70 system at a velocity of 0.0008 Å/ps and an elastic coefficient of 10, 20, 30 and 40 kcal/mol·A^{-2}.

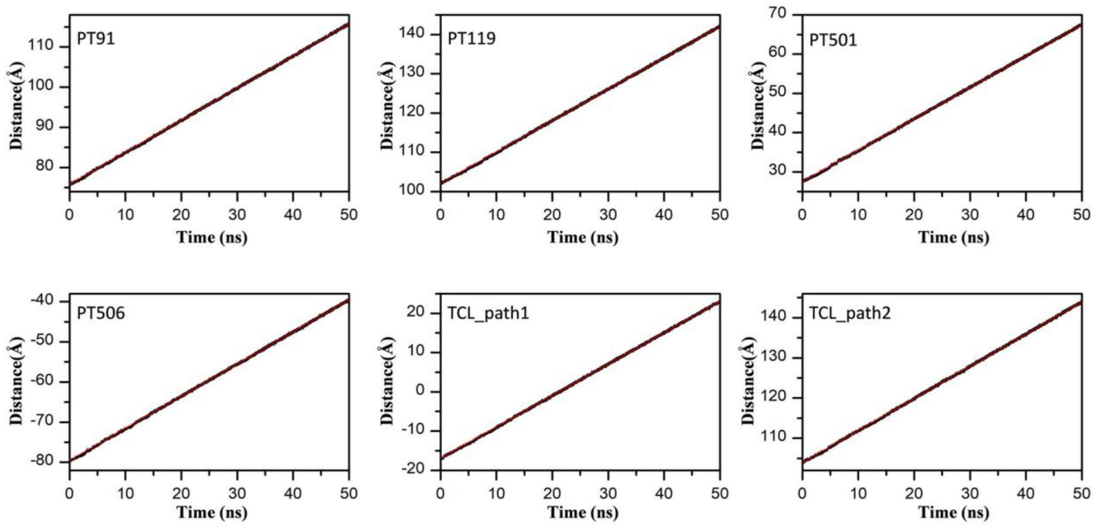

Figure S6. Variation of the distance between the ligand and the center of mass of the binding pocket versus simulation time for other systems at a velocity of 0.0008 Å/ps and an elastic coefficient of 20 kcal/mol·A^{-2}.

Table S1. Statistics of dissociation pathways for each inhibitor

	TCL	PT70	PT91	PT119	PT501	PT506
path1	50	95	104	2	98	27
path2	69	25	16	102	22	91
path3	1	0	0	16	0	2
